# “… wise, amazed, temp’rate, and furious, Loyal and neutral, in a moment”: first heritability analysis of affective temperaments reports remarkably high SNP-based heritability

**DOI:** 10.1192/j.eurpsy.2023.768

**Published:** 2023-07-19

**Authors:** X. Gonda, D. Torok, N. Eszlari, D. Gyorik, A. Millinghoffer, G. Bagdy, G. Juhasz

**Affiliations:** 1 Department of Psychiatry and Psychotherapy, Semmelweis University; 2 NAP3.0 Neuropsychopharmacology Research Group, Hungarian Brain Research Program, Semmelweis University; 3 Department of Pharmacodynamics, Semmelweis University; 4 Department of Measurement and Information Systems, Budapest University of Technology and Economics; 5 SE-NAP 2 Genetic Brain Imaging Migraine Research Group, Hungarian Brain Research Program, Semmelweis University, Budapest, Hungary

## Abstract

**Introduction:**

Depression shows a moderate heritability of 37-42%, which can be up to 75% in severely depressed samples 75%. At the same time SNP-based heritability of depression in GWAS-s is around 8-9%. Heterogeneity of the depressive phenotype may contribute not only to the lack of understanding its genetic background but may also hinder the identification of novel targets. Thus clinically relevant intermediate endophenotypes are needed for. The affective temperaments in the Akiskal model may be considered high-risk states or subclinical manifestations of mood disorders. Considering their strong genetic and biological background, high heritability in family studies, and their temporal stability, they may prove to be relevant endophenotypes for depression.

**Objectives:**

The aim of the current study was to investigate the genetic determinants and heritability of affective temperaments based on a GWAS approach.

**Methods:**

775 subjects aged between 18-60 years recruited in Budapest, Hungary provided genetic samples and completed questionnaires including the TEMPS-A (Temperament Evaluation of Memphis, Pisa, Paris and San Diego) scale. A genome-wide association analysis was performed with the five affective temperaments as outcome variables. Age, gender, the top 10 principal components of the genome, and the other 4 phenotype were added in the model as covariates. Summary statistics derived from the GWAS analyses were used to estimate the heritability, i.e. the genetic variance explained by the different affective temperaments. LD score regression using LDPred2 [4] was performed to estimate heritability from the beta values and effect size in case of all 5 affective temperament phenotypes.

**Results:**

rs3798978 showed a genome-wide significance (p=4.44x10-8) for anxious temperament, and several other variants showed suggestive significances for all five temperaments. The highest estimated heritability (h2 = 0.5224) was observed for the depressive temperament, and similarly high heritability was observed for the hyperthymic temperament (h2 = 0.4956). Anxious and cyclothymic temperaments showed almost the same heritability (cyclothymic h2 = 0.1651, anxious h2 = 0.1663), whereas for the irritable temperament, we got negative heritability estimation (h2 = -0.0567), which means that all of the phenotypic variance is explained by environmental factors.

**Conclusions:**

Our analyses yielded remarkably high heritability values for depressive and hyperthymic temperaments explaining 52% and 50% of phenotypic variances. In contrast to the 8-9% SNP-based heritability in depression studies our findings suggest that these temperaments may be relevant endophenotypes for mood disorders.

**Disclosure of Interest:**

None Declared

